# PermuteDDS: a permutable feature fusion network for drug-drug synergy prediction

**DOI:** 10.1186/s13321-024-00839-8

**Published:** 2024-04-15

**Authors:** Xinwei Zhao, Junqing Xu, Youyuan Shui, Mengdie Xu, Jie Hu, Xiaoyan Liu, Kai Che, Junjie Wang, Yun Liu

**Affiliations:** 1 Department of Medical Informatics, School of Biomedical Engineering and Informatics, Nanjing Medical University, 101 Longmian Avenue, Nanjing, 211166 Jiangsu China; 2 The Second Clinical Medical School, Nanjing Medical University, 101 Longmian Avenue, Nanjing, 211166 Jiangsu China; 3 Institute of Medical Informatics and Management, Nanjing Medical University, 101 Longmian Avenue, Nanjing, 210029 Jiangsu China; 4 Department of Information, the First Affiliated Hospital, Nanjing Medical University, No. 300 Guang Zhou Road, Nanjing, 210029 Jiangsu China; 5 Faculty of Computing, Harbin Institute of Technology, No. 92 West Da Zhi St, Harbin, 150001 Heilongjiang China; 6 Xi’an Aeronautics Computing Technique Research Institute, AVIC, No. 156, TaiBai Nroth Road, Xi’an, 710068 Shanxi China; 7 Aviation Key Laboratory of Science and Technology on Airborne and Missleborne Computer, Xi’an, 710065 Shanxi China

**Keywords:** Drug combination, Permutable feature fusion, Synergistic effect, Cancer

## Abstract

**Motivation:**

Drug combination therapies have shown promise in clinical cancer treatments. However, it is hard to experimentally identify all drug combinations for synergistic interaction even with high-throughput screening due to the vast space of potential combinations. Although a number of computational methods for drug synergy prediction have proven successful in narrowing down this space, fusing drug pairs and cell line features effectively still lacks study, hindering current algorithms from understanding the complex interaction between drugs and cell lines.

**Results:**

In this paper, we proposed a Permutable feature fusion network for Drug-Drug Synergy prediction, named PermuteDDS. PermuteDDS takes multiple representations of drugs and cell lines as input and employs a permutable fusion mechanism to combine drug and cell line features. In experiments, PermuteDDS exhibits state-of-the-art performance on two benchmark data sets. Additionally, the results on independent test set grouped by different tissues reveal that PermuteDDS has good generalization performance. We believed that PermuteDDS is an effective and valuable tool for identifying synergistic drug combinations. It is publicly available at https://github.com/littlewei-lazy/PermuteDDS.

**Scientific contribution:**

First, this paper proposes a permutable feature fusion network for predicting drug synergy termed PermuteDDS, which extract diverse information from multiple drug representations and cell line representations. Second, the permutable fusion mechanism combine the drug and cell line features by integrating information of different channels, enabling the utilization of complex relationships between drugs and cell lines. Third, comparative and ablation experiments provide evidence of the efficacy of PermuteDDS in predicting drug-drug synergy.

**Supplementary Information:**

The online version contains supplementary material available at 10.1186/s13321-024-00839-8.

## Introduction

Single-agent targeted therapies have been widely utilized in clinical cancer treatments. However, the clinical efficacy of monotherapy remains limited due to the biological complexity of tumors and the presence of pre-existing or acquired drug resistance mechanisms [1]. In contrast, combination therapies involving the simultaneous administration of multiple drugs have shown promising results in cancer treatment [2]. Compared to monotherapy, combination therapies have the potential to enhance treatment efficacy, reduce the dose-limiting toxicity associated with single agents, and overcome drug resistance [3]. Despite the potential benefits, it is important to note that not all drug combinations exhibit synergistic effects, and some combinations may even result in antagonistic interactions [4]. Therefore, it is essential to accurately determine the interaction between drug combinations to specific diseases.

Traditionally, the discovery of drug combinations heavily relied on clinical trials, posing challenges in terms of time consumption and cost-effectiveness due to the extensive number of potential drug combinations [5]. An alternative method is high-throughput screening (HTS), which enables automated testing of chemical and biological compounds against specific biological targets [6]. HTS techniques have significantly reduced the time required for identifying potential drug combinations. However, it is important to note that HTS has limitations in revealing the in vivo action modes of drug molecules [7]. Moreover, the substantial increase in the number of available drugs has rendered it impractical to comprehensively test the entire combinatorial space using HTS [8]. The sheer magnitude of potential drug combinations poses a challenge in terms of feasibility and resource utilization.

Computational methods, particularly machine learning models, have emerged as powerful tools for exploring the vast synergistic space of drug combinations [9]. These machine learning-based models can be broadly categorized into two types: classical machine learning and deep learning. Classical machine learning models, such as random forest [10], support vector machine (SVM) [11], extreme gradient boosting (XGBoost) [12], have been widely utilized in drug synergy prediction. These models leverage genomic information from cancer cells, physiochemical properties of drugs, and drug-cell interaction data to predict drug synergy [13]. For instance, Jeon et al. [14] employed random forest and SVM algorithms to predict the synergistic effects of anticancer drug combinations by incorporating monotherapy response and synthetic lethality information. However, this approach heavily relies on the given dataset, and the model may struggle to accurately predict the features of drug combinations without prior knowledge. Another study by He et al. [15] proposed an XGBoost-based model that predicts the selective synergistic effects of cancer by utilizing distinct single compound sensitivity curves between patient cells and healthy controls. This approach aimed to minimize potential toxicity associated with drug combinations.

Recently, deep learning has gained increasing popularity in drug development and discovery. Compare to classical machine learning methods, deep learning algorithms have better generalization performance, making it efficient in handling large drug combination datasets. Deep learning-based methods typically frame the challenge of drug synergy prediction as a regression task, aiming to predict the quantitative synergy scores. These methods can be categorized into three groups: fingerprint-based methods, SMILE-based methods and graph-based methods. Fingerprint-based methods take molecular fingerprint (also called descriptor) as input. Preuer et al. [9] proposed DeepSynergy, a feedforward neural network-based deep learning model for drug synergy score prediction that employs molecular fingerprints and gene expression as inputs. The performance of DeepSynergy demonstrated significant improvement over classical machine learning models. Kuru et al. [16] developed a MatchMaker model for predicting synergistic drug combinations using chemical descriptors generated by ChemoPy [17] and the expression profiles of landmark genes as input. Lin et al. [18] amalgamated molecular fingerprint information with drug induced gene expression profiles to capture drug cell responses to reveal the mechanisms of biological synergistic effects, and using deep forests as feature learning models. Hosseini et al. [19] proposed CCSynergy, used a feed forward network to obtain the fusion feature of drug fingerprints and mutiple cell line representations for durg syergy prediction. SMILE-based methods select simplified molecular-input line-entry system (SMILES) [20] as drug representations. Kim et al. [21] utilized a muti-head attention mechanism and convolutional neural networks (CNN) to encode drug SMILES. Graph-based methods that extract features from molecular graphs have the potential to capture structural information about the molecules [22]. Wang et al. [23] proposed DeepDDS, a model that utilizes a graph convolutional network (GCN) and attention mechanism to compute drug embedding vectors, which are integrated with cell line gene expression data to predict drug synergy. Liu et al. [24] proposed HypergraphSynergy, a method employing hypergraph representation learning to predict anti-cancer drug synergy by considering drugs and cell lines as nodes and representing drug pair-cell line triplets as hyperedges.

The previously mentioned deep learning-based methods have exhibited impressive performance in predicting drug synergy, yet there is still room for further improvement. One limitation is that these methods typically only use single type drug representation, thereby failing to provide a comprehensive description of drugs. Moreover, most of these methods simply fuse drug and cell line features through simple operations such as concatenation or addition, neglecting dynamic synergies between drug pairs and cell lines.

In light of the limitations associated with existing approaches for predicting drug synergy, we proposed a Permutable feature fusion network for Drug-Drug Synergy prediction, namely PermuteDDS. Our method addresses the challenge of limited representation types by considering multiple representations of drugs and cell lines. We employed the one-dimensional CNN to learn and extract high-latent features from these representations. Then, we designed a permutable fusion mechanism to combine the drug and cell line features, allowing us to capture the complex relationships between drug combinations and cell lines. Finally, we conducted experiments on two benchmark datasets, and the results compared to state-of-the-art methods indicated that PermuteDDS is an effective model for drug synergy prediction. In summary, the contributions of our work are as follows:

- We propose PermuteDDS, a deep learning model for drug-drug synergy prediction with multiple input representations.
- Our major contribution lies in the application of the permutable fusion mechanism to represent the interactions between drug combinations and cell lines.
- We conduct comprehensive experiments to demonstrate the effectiveness of our proposed model. We present ablations and analysis to gain a deeper understanding of the model’s behavior.

## Materials and methods

### Data description

We collected Drug-Drug Synergy (DDS) data, molecular structures of drugs, the expression profiles and mutation information of cell lines from various public databases as below.

- **Drug-Drug Synergy datasets.** The Drug-Drug Synergy (DDS) data were obtained from two released large-scale oncology screening datasets, namely O’Neil [25] and NCI-ALMANAC [26]. The O’Neil dataset contains 22 737 samples estimated by Loewe synergy score [27] consisting of two chemicals and a cell line, covering 38 unique drugs and 39 diverse caner cell lines. The NCI-ALMANAC dataset comprises 304 549 samples evaluated by the ComboScores of pairwise combinations of 104 FDA-approved anticancer drugs against a panel of 60 well-characterized human tumor cell lines.
- **Drug synergy scores.** The Loewe synergy score incorporates the concepts of sham combination and dose equivalence. Let *A* and *B* denote two drugs, and $x_A$ and $x_B$ represent their respective doses. The drug synergy score can then be computed as follows [27]:
  1 $$\begin{aligned} Y_{Loewe}=\frac{R_{\min }+R_{\max }\left( \frac{x_A+x_B}{m}\right) ^\lambda }{1+\left( \frac{x_A+x_B}{m}\right) ^\lambda } \end{aligned}$$
  where $Y_{Loewe}$ is a continuous value, $R_{\min }$ and $R_{\max }$ represents the maximum and minimum drug response, respectively. $\lambda$ is the shape parameter and *m* is the dose that produce midpoint between $R_{\min }$ and $R_{\max }$. The determination of combination benefit, denoted as ‘ComboScore’ [26], relies on a modification of Bliss independence [28]. Let $Y_{i}^{A_p B_q}$ represent the growth fraction for the *i*-th cell line exposed to the *p*-th concentration of drug A and the *q*-th concentration of drug B. Similarly, let $Z_i^{A_p B_q}$ denote the expected growth fraction for the combination. The final continuous combination score $Y_{ComboScore}$ for the cell line and the drug combination can then be computed as the sum of the differences between expected and observed growth fractions:
  2 $$\begin{aligned} Y_{ComboScore}=\sum _{p, q} Y_i^{A_p B_q}-Z_i^{A_p B_q} \end{aligned}$$
- **Molecular structures of drugs.** The SMILES of the drugs were obtained from PubChem database [29], based on which the chemical and structure information of a drug can be converted to fingerprints with RDKit [30] and MAP4 project.^1^
- **Gene expression and gene mutation of cell lines.** The gene expression and gene mutation of cell lines were obtained from Genomics of Drug Sensitivity in Cancer (GDSC) database,^2^. where 1000 human cancer cell lines were characterised and screened [31]. To enhance the representation of cell lines, we identified significant genes by referencing The Library of Integrated Network-Based Cellular Signatures (LINCS) project [32]. The LINCS project offers a meticulously curated set of approximately 1000 genes, known as the ‘landmark gene set’, which captures 80% of the information from Connectivity Map data [33]. We selected genes that intersected between GDSC gene expression profiles and the landmark set, as well as gene mutation profiles. Finally, 899 genes were chosen for expression profiles, and 968 genes were selected for mutation profiles.

We preprocessed the two dataset by eliminating the drugs lacking SMILES and the cell lines devoid of gene expressions or gene mutations. The resulting refined O’Neil dataset comprised 18 950 samples with Loewe synergy scores across 38 drugs and 39 cell lines, while the processed NCI-AlMANAC dataset consisted of 74 139 instances with NCI ComboScores (a Bliss independence modification) across 87 drugs and 55 cell lines.

### Molecular fingerprints

Molecular fingerprints (also referred to as descriptors) is a prevalent method of drug representation. Within this approach, each molecule is encoded as a binary string that delineate the presence or absence of specific substructure fragments or properties within the molecule. A value of 0 corresponds to absence of the particular feature, while a value of 1 denotes its existence. Moreover, the fingerprints exhibit uniqueness; specifically, one molecule possesses one sequence for a particular fingerprints type, yet it can have numerous diverse fingerprints types [22]. The three different kinds of fingerprints employed in this article are described below.

- **Hashed topological-torsion (HashTT)** is a kind of topological fingerprints [34]. The fragments employed in HashTT fingerprints are linear sequence comprising four consecutively bonded non-hydrogen atoms. The label for each fragment is determined based on its atomic type, the number of non-hydrogen branches attached to it, and its number of 7r electrons of each atom. These labels then undergo a hashing process to generate the HashTT fingerprints.
- **MinHashed atom-pair fingerprint up to a diameter of four bonds (MAP4)** [35] is a novel fingerprint that integrates both substructure fingerprints and atom-pair fingerprints. Given a molecule SMILE sentence, the circular substructures with radii of r = 1 and r = 2 bonds around individual atoms within an atom-pair configuration are encoded as two pairs of SMILES. Each pair is subsequently combined with the topological distance separating the central atoms. These atom-pair molecular shingles undergo a hashing process, and the resulting set of hashes is subjected to the MinHashed technique, which lead to the creation of the MAP4 fingerprint.
- **Molecular ACCess System (MACCS)** [36] keys are one of the most commonly used structural fingerprints. In this fingerprint, one molecule is encoded as a 166-bit binary string, where each bit corresponds to a predefined structural fragment (e.g. 3-element ring).

**Fig. 1:**
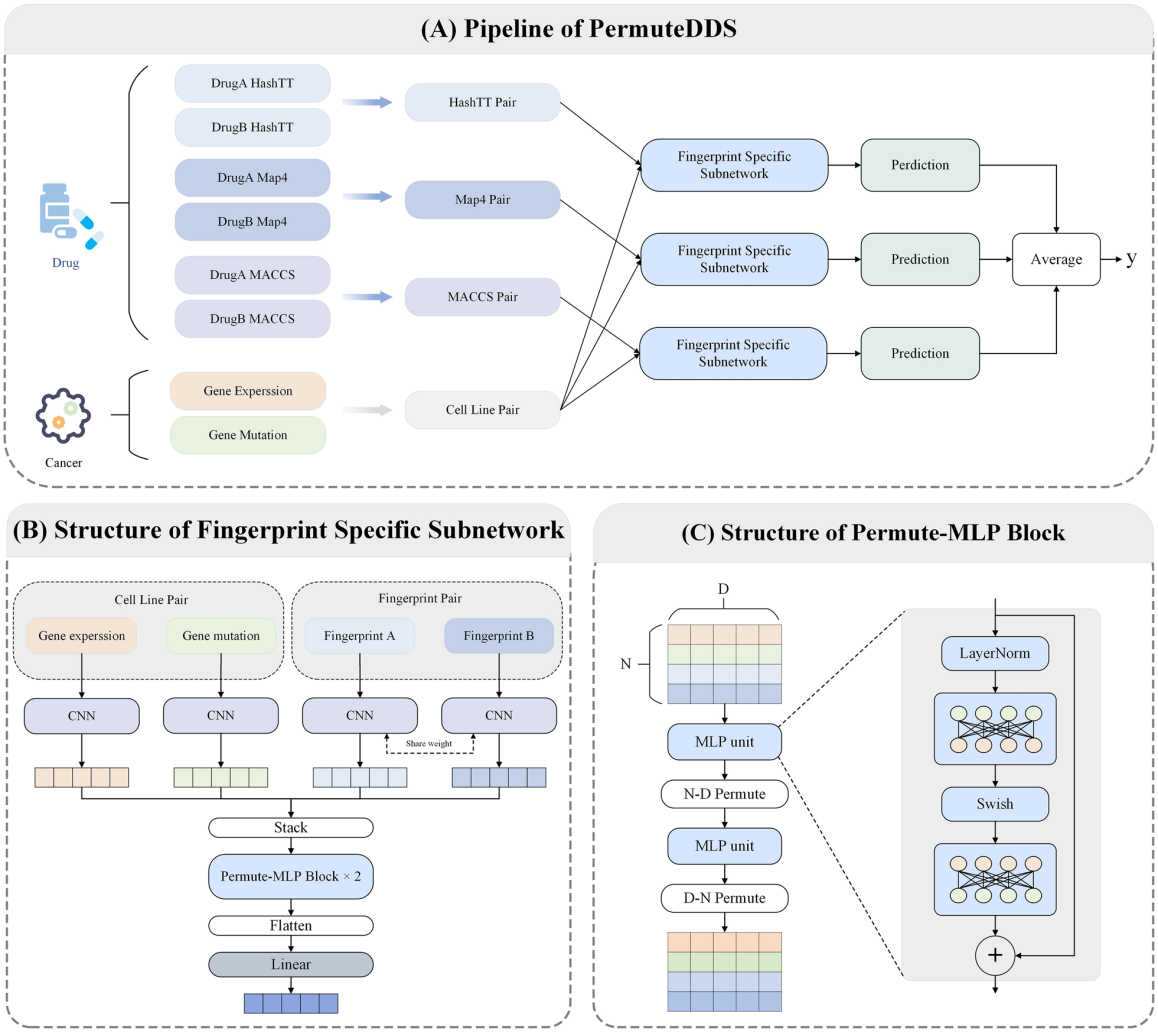
The overview of the PermuteDDS framework

### Overview of PermuteDDS

PermuteDDS is an end-to-end framework for predicting drug combinations, presented in Fig. 1. Specifically, Fig. 1(A) illustrates the pipeline implemented by PermuteDDS to derive the synergy scores. Our framework contains three subnetworks called Fingerprint Specific Networks (FSNs), all of which share the same architecture shown in Fig. 1(B). Each FSN takes a drug pair of a certain fingerprint and a cell line pair including its gene expression and gene mutation profiles as inputs. Then, the CNNs are used to extract drug and cell line features, and the Permute-MLP module (Fig. 1(C)) is proposed to obtain the fusion synergy feature, which is the output of FSN. The three FSNs output three distinct synergy features, each of which is propagated through a specific prediction module to generate a synergy score. The final outputs of PermuteDDS is the average of the synergy scores predicted by these prediction modules.

### Fingerprint specific subnetwork (FSN)

Given a drug fingerprint pair and a cell line pair as inputs, the FSN encompasses three essential processes, including drug features extraction, cell line features extraction and feature fusion, which integrates the extracted drug and cell line features. The output of FSN is a synergy feature. A comprehensive overview of these processes is provided below.

**Fig. 2:**
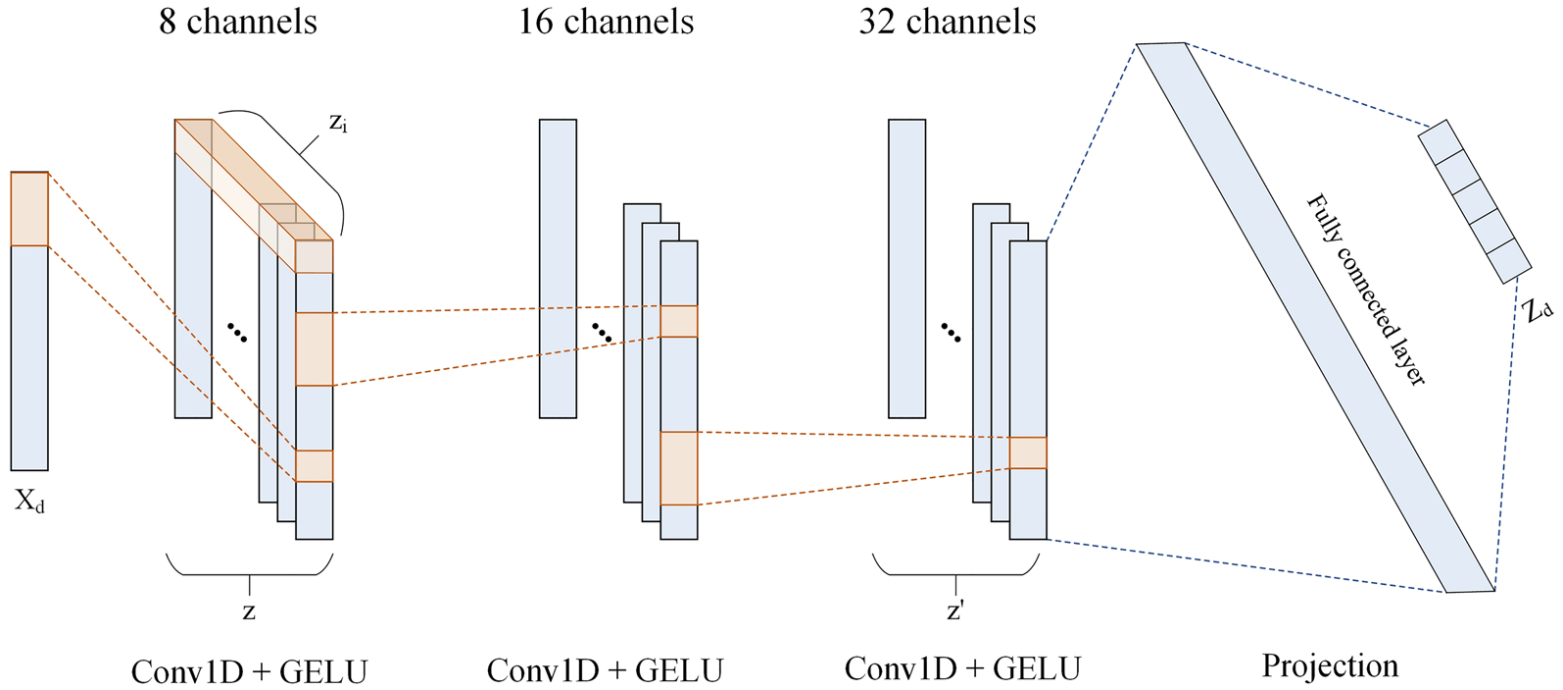
The architecture of the three-layer Conv1D

**Drug features**   To extract the drug fingerprint features, we used the one-dimensional convolutional neural network (Conv1D), where 1D array-like kernel convolves along a single dimension and identify the patterns from fingerprint information. Here, we considered three different fingerprints include Hashtt(*t*), MAP4(*p*) and MACCS(*s*). We conducted a three-layer Conv1D model, as illustrated in Fig. 2. An input fingerprint sequence is represented as ${\textbf{X}}_d\in {\mathbb {R}}^{L\times 1}$ in which *L* denotes the length, 1 denotes the dimension and $d \in \{t, p, s\}$. Let ${\textbf{X}}_{i: i+j}$ refer to the concatenation of tokens ${\textbf{X}}_i, {\textbf{X}}_{i+1},..., {\textbf{X}}_{i+j}$ in the sequence, a feature $z_i\in {\mathbb {R}}^{k}$ can be generated from the window of tokens ${\textbf{X}}_{i: i+h-1}$ by

3 $$\begin{aligned} z_i=\textrm{GELU}\left( {\textbf{W}}_k \cdot {\textbf{X}}_{i: i+h-1}+b_k\right) , \end{aligned}$$

where $\textrm{GELU}$ [37] is an activation function, ${\textbf{W}}_k\in {\mathbb {R}}^{h\times 1 \times k}$ is a *k*-channel (here is 8) filter applied to a window of *h* token, $b_k\in {\mathbb {R}}$ is a bias term and $z_i\in {\mathbb {R}}^{k}$ is a feature generated from the window of tokens ${\textbf{X}}_{i: i+h-1}$. Then, the filter is applied to each possible window of tokens to produce a feature map

4 $$\begin{aligned} {\textbf{z}} = \textrm{Concat}(z_1, z_2,..., z_L), \end{aligned}$$

where $\mathrm Concat$ refers to the concatenation process and ${\textbf{z}}\in {\mathbb {R}}^{L\times k}$. Subsequently, we conducted similar operations as above with different filter channels (16 and 32 respectively) in the next layers and then got an output $\mathbf {z'}\in {\mathbb {R}}^{L\times k'}$ in which ${k'}$ denote the output channel (here is 32) of the third layer. To obtained the final drug features with expected dimension *D*, we conducted a projection process as follow:

5 $$\begin{aligned} {\textbf{Z}}_d = {\textbf{W}}_d \cdot \textrm{Flatten}(\mathbf {z'}) + b_d, \end{aligned}$$

where $\mathrm Flatten$ refers to the flattening process, ${\textbf{W}}_d\in {\mathbb {R}}^{Lk'\times D}$ is the weight matrix, $b_d$ is the bias term and ${\textbf{Z}}_d\in {\mathbb {R}}^{D}$ is the output drug features. Then, given a drug pair (*i*, *j*), the pairwise features can be represented as $({\textbf{Z}}_{di}, {\textbf{Z}}_{dj})$, where ${\textbf{Z}}_{di}\in {\mathbb {R}}^{D}$ and ${\textbf{Z}}_{dj}\in {\mathbb {R}}^{D}$.

**Cell line features**   To derive cell line features, we employed two distinct three-layer CNNs individually for gene expression (*e*) and gene mutation (*m*) data. Consistent with the fingerprint sequence, an initial gene expression sequence can be represented as ${\textbf{X}}_{e}\in {\mathbb {R}}^{l\times 1}$ in which *l* denotes the number of selected genes and 1 is the dimension. We then conducted the same operations described in the Eq. 3, 4 and 5 to obtained the gene expression features ${\textbf{Z}}_{e}\in {\mathbb {R}}^{D}$ with *D* represents the output dimension, which aligns with the dimension of drug features. Similarly, the extracted gene mutation features are represented as ${\textbf{Z}}_{m}\in {\mathbb {R}}^{D}$.

**Feature fusion**   Recent works [38, 39] have demonstrated the superior performance of Multilayer Perceptron (MLP) in feature fusion tasks. Motivated by these findings, we conducted a MLP-based method to extract fusion feature of drug fingerprints and cell lines. Given a triplet of drug pairs and cell line, a feature ${\textbf{H}}_d \in {\mathbb {R}}^{N\times D}$ is constructed by

6 $$\begin{aligned} {\textbf{H}}_d = \textrm{Stack}({\textbf{Z}}_{di}, {\textbf{Z}}_{dj}, {\textbf{Z}}_{e}, {\textbf{Z}}_{m}). \end{aligned}$$

Here, *N* corresponds to the value 4, reflecting the number of features utilized in the stacking process. We then employed two Permute-MLP blocks taken the feature $\mathbf {H_d}$ as input. A diagrammatic illustration of the Permute-MLP block is presented in Fig. 1(C). First, we conducted a MLP unit along the *D*-channel, which can be formulated as follows:

7 $$\begin{aligned} \widehat{\textbf{H}}_d={\textbf{H}}_d+\textrm{LN}(\textrm{Swish}({\textbf{H}}_d{\textbf{W}}_{dD}^1){\textbf{W}}_{dD}^2), \end{aligned}$$

where $\textrm{Swish}$ [40] is an activation function, $\textrm{LN}$ refers to LayerNorm [41], ${\textbf{W}}_{dD}^1, {\textbf{W}}_{dD}^2\in {\mathbb {R}}^{D\times D}$ are the weight matrices and $\widehat{\textbf{H}}_d\in {\mathbb {R}}^{N\times D}$. Then, we perform a $N\text{- }D$ permutation operation with respect to $\widehat{\textbf{H}}_d$, yielding $\widehat{\textbf{H}}_d^{\top }\in {\mathbb {R}}^{D\times N}$, which is serve as the input to the next MLP unit along the *N*-channel. This process can be described as

8 $$\begin{aligned} \overline{\textbf{H}}_d = \widehat{\textbf{H}}_d^{\top }+\textrm{LN}(\textrm{Swish}(\widehat{\textbf{H}}^{\top }_d{\textbf{W}}_{dN}^1){\textbf{W}}_{dN}^2), \end{aligned}$$

where $\overline{\textbf{H}}_d\in {\mathbb {R}}^{D\times N}$, ${\textbf{W}}_{dN}^1\in {\mathbb {R}}^{N\times D}$ and ${\textbf{W}}_{dN}^2\in {\mathbb {R}}^{D\times N}$. To recover the original dimensional information to $\mathbf {H_d}$, we only need to perform a $D\text{- }N$ permutation operation on $\overline{\textbf{H}}_d$, outputting $\overline{\textbf{H}}_d^{\top }\in {\mathbb {R}}^{N\times D}$, which is the input to the next Permute-MLP block. Similarly, we conducted the same operations as above in the second block and obtained a fusion feature $\widetilde{\textbf{H}}_d\in {\mathbb {R}}^{N\times D}$. Furthermore, we conducted projection process to obtain the final synergy feature

9 $$\begin{aligned} {\textbf{M}}_d=\textrm{GELU}(\textrm{Flatten}(\widetilde{\textbf{H}}_d){\textbf{W}}_{dproj}^1){\textbf{W}}_{dproj}^2, \end{aligned}$$

where ${\textbf{W}}_{dproj}^1\in {\mathbb {R}}^{ND\times \widehat{D}}$ with hidden size $\widehat{D}$, ${\textbf{W}}_{dproj}^2\in {\mathbb {R}}^{\widehat{D}\times \widetilde{D}}$ with the output dimension $\widetilde{D}$, and ${\textbf{M}}_d\in {\mathbb {R}}^{\widetilde{D}}$ is the output of $\textrm{FSN}_d$.

### Predicting the synergistic effect

The objective of drug synergy prediction is to derive a synergy score for a given drug pair and cell line trio. The process outlined above results in the generation of three distinct synergy features, denoted as ${\textbf{M}}_t$, ${\textbf{M}}_p$ and ${\textbf{M}}_s$, through the utilization of the respective modules $\textrm{FSN}_t$, $\textrm{FSN}_p$, and $\textrm{FSN}_s$. Each of the generated features was then propagated to a specific prediction module, which consisted of two linear transformations with a GELU activation in between, and output a synergy score ${\hat{y}}_d\in {\mathbb {R}}$. In other words, we got three different synergy scores denoted as ${\hat{y}}_t$, ${\hat{y}}_p$ and ${\hat{y}}_s$. Given a drug-drug-cell line trio, the final predicted synergy score ${\hat{y}}$ can be calculated as

10 $$\begin{aligned} {\hat{y}} = \frac{1}{3}({\hat{y}}_t+{\hat{y}}_p+{\hat{y}}_s) \end{aligned}$$

Then, the mean square error (MSE) is adopted as the loss function to train the model, which is defined as:

11 $$\begin{aligned} {\mathcal {L}}_{MSE} = \frac{1}{n} \sum _{i=1}^n\left( y_i-{\hat{y}}_i\right) ^2, \end{aligned}$$

where *n* represents the number of training set and $y_i$ is the real score of a given trio.

### Experimental setup

#### Data split strategies

**Fig. 3:**
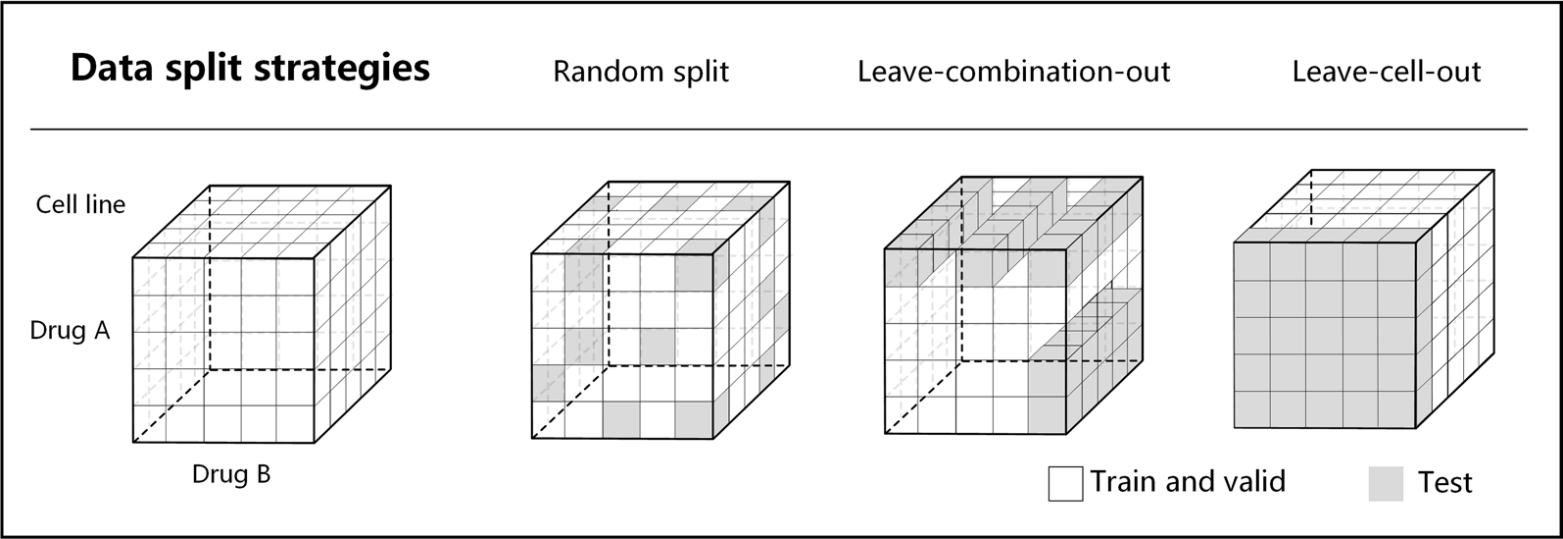
Three different data split strategies. The white parts are used as training and validation data. The grey parts indicate testing data

We first randomly selected 90% samples from each dataset to conduct a 5-fold cross-validation strategy. To benchmark the performance of graph-fp under different situations, we considered three different strategies shown in Fig. 3:

- **Random-split**. The training samples were randomly partitioned into five equal folds. Four of these folds were utilized as the training set, while the remaining one was designated as the test set.
- **Leave-cell-out**. The cell line set was randomly partitioned into five equal folds. Samples containing the cell lines in four of these folds were used as the training set, while the remaining samples were assigned to the test set. This ensured that the test set contained only cell lines that were not included in the training set.
- **Leave-combination-out**. The set of drug combinations was randomly partitioned into five equal folds. Samples containing the drug combinations in four of these folds were used as the training set, while the remaining samples were used as the test set, ensuring that the test set only contained unseen drug combinations that were not present in the training set.

Furthermore, to verify the generalization ability of our method, we used the remaining 10% samples as the independent test set. The models were first trained and evaluated on the cross validation set, and then tested on the independent test set.

#### Baselines

To evaluate the performance of our model, we compared it with several competitive deep learning methods.

- **HypergraphSynergy** [24] employed Hypergraph Neural Networks (HGNN) to predict drug synergy with hypergraphs as input. In this method, drugs and cell lines are represented as nodes, while synergistic drug-drug-cell line triplets are represented as hyperedges. We reproduced the results of HypergraphSynergy and obtained the remaining results as reported in the HypergraphSynergy paper.
- **DeepSynergy** [9] takes molecular chemistry and cell line gene expression as input and used a feed forward neural network to predict synergy scores.
- **DTF** [42] utilized a tensor factorization to decompose drug synergy matrix and the results of the tensor decomposition are used as features to train the DNN model for drug synergy prediction.
- **CombFM** [43] used a higher-order factorization machine to predict synergy scores with a higher-order tensor as input which is compiled by drugs, drug concentrations and cell lines.
- **Celebi’s method** [44] integrate drug synergy features with multiple biological and chemical properties’ features and employed an XG-Boost model to predict synergy scores.

#### Evaluation metrics

We regarded the drug synergy prediction as a regression task, which objective is to predict the quantitative synergy scores of drug combinations. The regression results were evaluated by three metrics: the root man squared error (RMSE), the coefficient of determination ($\textrm{R}^2$) and Pearson’s Correlation Coefficient (PCC). These evaluation metrics are calculated as follows:

12 $$\begin{aligned} \textrm{RMSE}= & {} \sqrt{\frac{\sum \limits _{i=1}^n\left( y_t^i-y_p^i\right) ^2}{n}} \end{aligned}$$

13 $$\begin{aligned} \textrm{R}^2= & {} 1-\frac{\textrm{RMSE}^2}{{\text {Var}}\left( y_t^i\right) },\quad \textrm{ where } {\text {Var}}\left( y_t\right) =\sum \limits _{i=1}^n\left( y_t^i-\bar{y}_t\right) ^2 \end{aligned}$$

14 $$\begin{aligned} \textrm{PCC}= & {} \frac{\sum \limits _{i=1}^n\left( y_t^i-\bar{y}_t\right) \left( y_p^i-\bar{y}_p\right) }{\sqrt{\sum \limits _{i=1}^n\left( y_t^i-\bar{y}_t\right) ^2} \sqrt{\sum \limits _{i=1}^n\left( y_p^i-\bar{y}_p\right) ^2}} \end{aligned}$$

In the equations, $y_t$ and $y_p$ denotes the true synergy scores and predicted synergy scores, respectively. $\bar{y}_t$ and $\bar{y}_p$ represent the mean value of the true synergy scores and predicted synergy scores, respectively.

## Results

### Performance comparison on cross validation

The 5-fold cross validation results of PermuteDDS and other competitive methods on O’Neil dataset are shown in Table 1. It can be easily seen that our PermuteDDS surpassed all baselines by a large margin on the random split task, e.g. 9.4% relative $\textrm{R}^2$ increase compared to the previous state-of-art method. Compared with random split task, leave-cell-out and leave-combination-out are more challenging, which test the performance of prediction models on unseen drug combinations/cell lines. On these two tasks, the performance of all methods decreased significantly compared to random split task. PermuteDDS performed slightly worse than HypergraphSynergy on the leave-cell-out task. This may be attributed to the fact that HypergraphSynergy utilized an auxiliary task that involved reconstructing cell line and drug similarity networks, which can enhance the robustness of the model on leave-cell-out task. However, this auxiliary task took all drugs and cell lines as input, resulting in the presence of unknown combinations and cell lines solely within the prediction module. Despite these considerations, the performance gap between our method and HypergraphSynergy remains quite small. As for the leave-combination-out task, PermuteDDS achieved the best results, with 19.3% relative $\textrm{R}^2$ increase and 8.1% relative PCC increase compared to HypergraphSynergy. These results highlight the superior predictive capabilities of PermuteDDS in accurately estimating drug synergy.

Table 2 shows that the performance of the models is comparatively lower on the NCI-ALMANAC dataset in comparison to the O’Neil dataset. This observation could be attributed to the fact that the NCI-ALMANAC dataset encompasses a wider range of drugs and cell lines, thereby increasing the complexity of prediction. However, PermuteDDS still achieved better performance in most cases. Regarding the random split task, PermuteDDS demonstrated superior performance compared to other state-of-the-art approaches. It achieved a RMSE of 43.053, a $\textrm{R}^2$ of 0.527 and a PCC of 0.726. However, similar to the results on the O’Neil dataset, our method only achieved competitive results compared to other methods. However, in the leave-combination-out task, PermuteDDS outperformed all other baseline methods by a large margin, achieving the lowest RMSE of 51.58, the highest $R^{2}$ of 0.318 and the highest PCC of 0.569.

**Table 1:** Performance comparison on the O’Neil dataset. Bold values indicate the best performance

	Random split			Leave-cell-out			Leave-combination-out		
	RMSE	$\textrm{R}^2$	PCC	RMSE	$\textrm{R}^2$	PCC	RMSE	$\textrm{R}^2$	PCC
PermuteDDS	**13**.**721**	**0**.**641**	**0**.**801**	19.668	0.243	0.522	**16**.**152**	**0**.**501**	**0**.**709**
HypergraphSynergy	14.727	0.586	0.775	**19**.**537**	**0**.**252**	0.533	17.346	0.420	0.656
DeepSynergy	14.87	0.584	0.765	23.890	0.195	0.426	17.28	0.433	0.663
ComboFM	16.86	0.451	0.702	20.820	0.142	0.396	18.62	0.376	0.635
DTF	14.73	0.594	0.775	21.110	0.132	**0**.**535**	17.37	0.429	0.671
Celebi’s method	16.34	0.5	0.708	20.6	0.179	0.473	19.1	0.309	0.572

**Table 2:** Performance comparison on the NCI-ALMANAC dataset. Bold values indicate the best performance

	Random split			Leave-cell-out			Leave-combination out		
	RMSE	$\textrm{R}^2$	PCC	RMSE	$\textrm{R}^2$	PCC	RMSE	$\textrm{R}^2$	PCC
PermuteDDS	**43**.**053**	**0**.**527**	**0**.**726**	54.128	0.242	0.519	**51**.**58**	**0**.**318**	**0**.**569**
HypergraphSynergy	43.89	0.508	0.719	**53**.**398**	**0**.**273**	**0**.**538**	52.609	0.291	0.543
DeepSynergy	44.44	0.491	0.701	54.560	0.230	0.322	53.500	0.262	0.526
ComboFM	48.27	0.399	0.651	54.670	0.245	0.531	53.890	0.267	0.526
DTF	47.03	0.430	0.678	54.730	0.223	0.517	53.470	0.263	0.531
Celebi’s method	47.31	0.423	0.653	53.49	0.259	0.516	55.830	0.196	0.456

### Performance evaluation on independent test

**Fig. 4:**
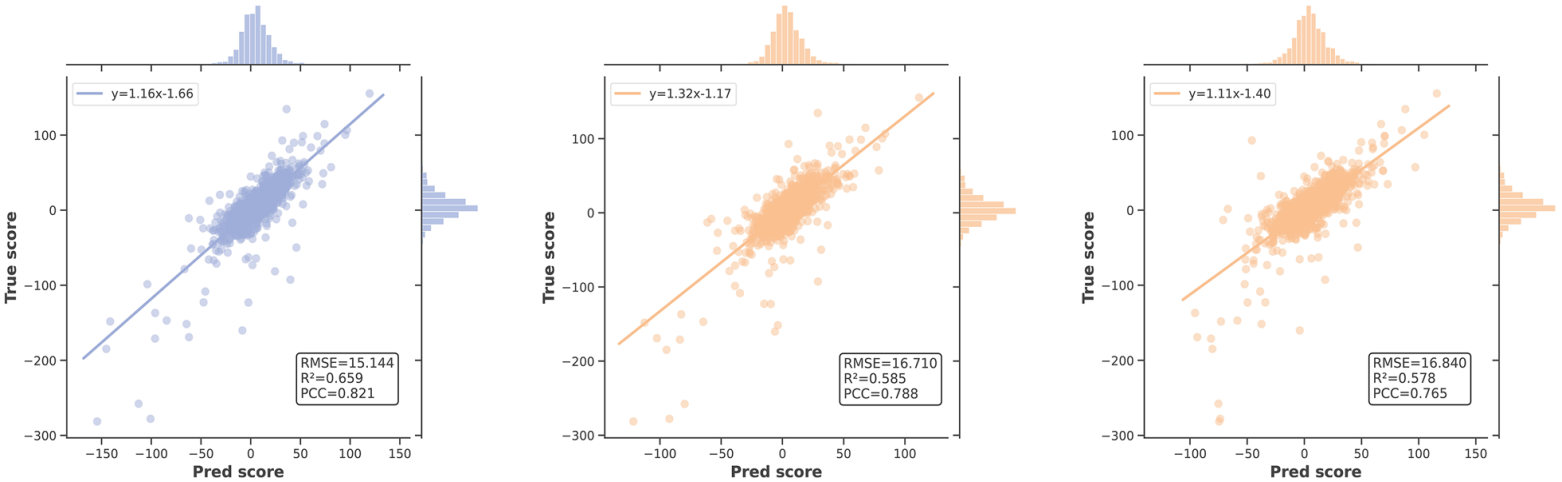
Independent test results on the O’Neil dataset. From left to right: PermuteDDS, HypergraphSynergy and DeepSynergy

**Fig. 5:**
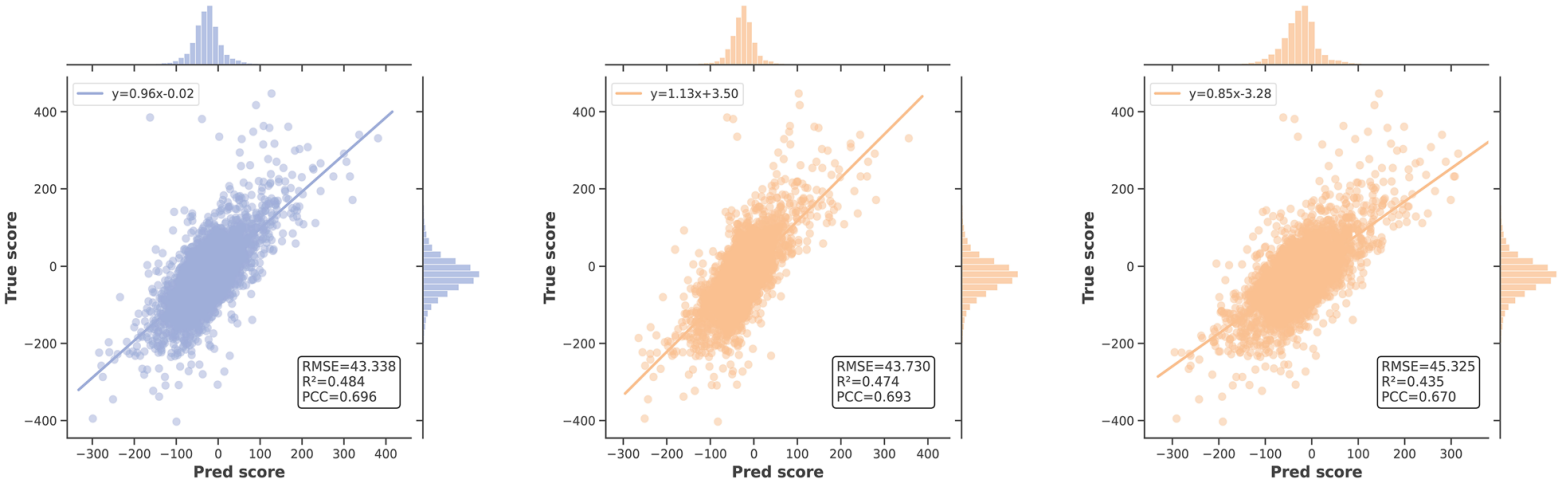
Independent test results on the O’Neil dataset. From left to right: PermuteDDS, HypergraphSynergy and DeepSynergy

Furthermore, we assessed the generalization performance of our model by testing on independent test sets, and the results are presented in Table 3. Notably, PermuteDDS achieved the overall best performance. Specifically, on the O’Neil dataset, PermuteDDS attained the top result with RMSE, $\textrm{R}^2$, and PCC scores of 15.144, 0.659, and 0.821, respectively. While the NCI-ALMANAC dataset’s complexity posed challenges for prediction across all methods, PermuteDDS still exhibited a slight superiority over previous state-of-the-art approaches with RMSE, $\textrm{R}^2$, and PCC scores of 43.338, 0.484, and 0.696, respectively. To intuitively assess differences in predictive performance across different datasets, we analyzed the distribution of true scores and predicted scores generated by the top three methods—PermuteDDS, HypergraphSynergy, and DeepSynergy. Figure 4 illustrates that the prediction results of all models on the O’Neil dataset form a well-clustered fitting line, indicative of good predictive performance. Conversely, as depicted in Fig. 5, the results on the ALMANAC datasets appear relatively dispersed, and the expansion of synergy scores (coordinate axis) range further confirms the complexity of this dataset.

**Table 3:** Performance comparison on the independent dataset. Bold values indicate the best performance

	O’Neil			NCI-ALMANAC		
	RMSE	R2	PCC	RMSE	R2	PCC
PermuteDDS	**15**.**144**	**0**.**659**	**0**.**821**	**43**.**338**	**0**.**484**	0.696
HypergraphSynergy	16.710	0.585	0.788	43.730	0.474	0.693
DeepSynergy	16.840	0.578	0.765	45.325	0.435	0.670
ComboFM	16.080	0.541	0.754	46.370	0.457	0.685
DTF	16.150	0.548	0.752	49.860	0.372	**0**.**700**
Celebi’s method	16.500	0.529	0.728	45.860	0.469	0.688

**Table 4:** Results of ablation study. Bold values indicate the best performance

	O’Neil			NCI-ALMANAC		
	RMSE	$\textrm{R}^{2}$	PCC	RMSE	$\textrm{R}^{2}$	PCC
PermuteDDS	**13**.**721**	**0**.**641**	**0**.**801**	**43**.**053**	**0**.**527**	0.726
w/o hashtt	13.895	0.632	0.796	44.081	0.504	0.711
w/o MAP4	13.746	0.639	0.800	43.964	0.507	0.713
w/o maccs	13.953	0.628	0.793	44.185	0.501	0.709
hashtt	14.340	0.607	0.780	46.034	0.459	0.681
maccs	14.444	0.602	0.777	46.141	0.456	0.681
MAP4	14.345	0.607	0.780	46.310	0.452	0.678
w/o Permute-MLP	14.811	0.581	0.769	44.528	0.494	0.718
PermuteDDS-L	14.433	0.602	0.777	43.138	0.525	**0**.**728**

### Ablation study

To study the effectiveness of each inputs and each PermuteDDS units, we perform the ablation studies under random split on both datasets. As shown in Table 4, the complete PermuteDDS framework achieves the best performance on 5 of 6 evaluation. In terms of the selection of input, we designed variants with different molecular fingerprint combinations as inputs. Upon examination, it becomes evident that the removal of any of the three fingerprints results in a decline in performance, and employing only one fingerprint yields even worse results. We can infer from the results that the combination of the three different fingerprints complements each other jointly contributes to the predictive performance of PermuteDDS. Then, in terms of model design, we conducted another two variants: PermuteDDS without Permute-MLP block and PermuteDDS-L. PermuteDDS-L is the model that utilizes a feedforward network to extract features from fingerprints and cell lines. The model without Permute-MLP block demonstrated inferior performance compared to PermuteDDS, highlighting the effectiveness of this module. Moreover, CNN might not capture as much information from fingerprints and cell lines as expected, as PermuteDDS-L only performed slightly worse than PermuteDDS.

### Model performance is robust against noise cell lines

**Fig. 6:**
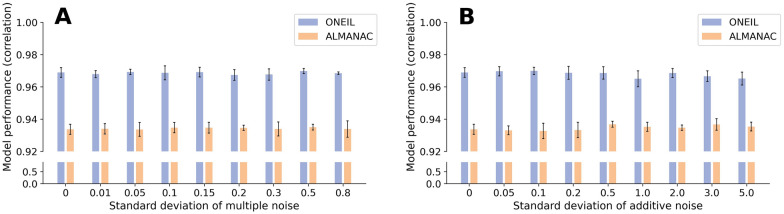
Correlation between predicted synergy scores of cell lines without noise and cell lines with multiplicative noise (A) or additive noise (B)

In actual clinical situations, cancer cells may be mixed with normal cells. To assess the robustness of PermuteDDS, we conducted a sensitivity analysis to evaluate the stability of model performance in response to noise, following the methodology outlined in [45]. Specifically, we introduced different levels of multiplicative or additive Gaussian noise into the input cell line gene expression and mutation profiles. Subsequently, PermuteDDS was trained using these resulting noisy cell lines. The underlying assumption is that Gaussian noise injected into the data can serve as a simulation of normal cell lines. For a given gene expression (or mutation) profile *X*, the computation of input noisy cell lines is as follows:

15 $$\begin{aligned} x_{mul}=\,& {} x * N\left( 1, \sigma _{mul}\right) \end{aligned}$$

16 $$\begin{aligned} x_{add}=\, & {} x + N\left( 0, \sigma _{add}\right) \end{aligned}$$

where $x_{mul}$ represent multiplicative noisy cell lines generated with a Gaussian distribution $N\left( 1, \sigma _{mul}\right)$, and $x_{add}$ represent additive noisy cell lines generated with a Gaussian distribution $N\left( 0, \sigma _{add}\right)$. We adopted the same standard deviation as reported in [45]. Subsequently, we performed 5-fold cross-validation under random split across each standard deviation on both datasets. As depicted in Fig. 6A, the predicted synergy scores, obtained by training on cell lines with multiplicative Gaussian noise, exhibit consistently high correlations with those trained on the original cell lines. Remarkably, even as the magnitude of the noise increases, the stability of the model’s performance is retained. Similar behavior is observed when subjecting PermuteDDS to additive Gaussian noise, as illustrated in Fig. 6B. Based on these observations, we can infer that PermuteDDS demonstrates robustness in the presence of noisy data.

### Cell line data study

Cell lines are known to exhibit sensitivity to variations in experimental conditions. Even identical cell lines obtained from different institutions can exhibit distinct gene expression profiles. To assess the necessity of the selected cell line descriptors, we constructed the variant of the PermuteDDS employing the cell line gene expression profiles sourced from the Cell Lines Project data in the COSMIC database [46]. Following the methodology outlined in [24], we considered only data related to 651 genes from the COSMIC Cancer Gene Census (https://cancer.sanger.ac.uk/census.). Moreover, we created an another variant utilizing a simple one-hot encoding as the input cell line descriptor, serving as a baseline for comparison. For the leave-cell-out task, the cell lines within the test set, which are unseen during training, are encoded as simple zero vectors. The results on the O’Neil and NCI-ALMANAC datasets are presented in Table 5 and Table 6, respectively. The term ‘cline-gdsc’ denotes the original PermuteDDS, whereas ‘cline-cosmic’ and ‘cline-onehot’ refer to the variants employing COSMIC and one-hot cell line descriptors, respectively. The outcomes derived from employing different cell lines descriptors consistently exhibit similar performance across all cross-validation scenarios. This suggests that our method demonstrates insensitivity towards the choice of these cell-line descriptors, as long as the representation method adequately represents and distinguishes different cell lines. It’s crucial to highlight that all variants exhibited poor performance under the leave-cell-out task. Even upon encoding an unseen cell line with a zero vector, no significant differences were observed in comparison to the utilization of gene expression profiles. Indeed, none of the baseline methods demonstrate the capability to achieve satisfactory results on this task (Tables 1 and 2), underscoring the considerable challenge inherent in its execution.

Furthermore, we conducted similar experiments using one-hot encoding as cell line descriptors to construct the other two baseline methods, DeepSynergy and HypergraphSynergy. As presented in Additional file 1: Tables S1 and S2, DeepSynergy exhibited a similar phenomenon to PermuteDDS, wherein employing one-hot encoding as a cell line descriptor yielded similar results to the original. However, replacing the cell line descriptor with one-hot encoding leads to a significant decline in the performance of HypergraphSynergy. This may be attributed to the fact that HypergraphSynergy incorporates the reconstruction of cell line similarities as part of its optimization objective. Since the similarity between cell lines encoded using one-hot encoding is uniformly zero, this reconstruction process loses its significance. These observations reveal the inconsistent sensitivity among different methods to the selection of cell line descriptors, depending on the preprocessing approach applied to the cell lines.

**Table 5:** Performance comparison of different cell lines descriptors on the O’Neil dataset

	Random split			Leave-cell-out			Leave-combination-out		
	RMSE	$\textrm{R}^2$	PCC	RMSE	$\textrm{R}^2$	PCC	RMSE	$\textrm{R}^2$	PCC
cline-gdsc	13.721	0.641	0.801	19.668	0.243	0.522	16.152	0.501	0.709
cline-cosmic	13.602	0.647	0.805	19.928	0.236	0.508	16.122	0.503	0.71
cline-onehot	13.663	0.644	0.803	20.010	0.232	0.507	16.175	0.499	0.708

**Table 6:** Performance comparison of different cell lines descriptors on the NCI-ALMANAC dataset

	Random split			Leave-cell-out			Leave-combination-out		
	RMSE	$\textrm{R}^2$	PCC	RMSE	$\textrm{R}^2$	PCC	RMSE	$\textrm{R}^2$	PCC
cline-gdsc	43.053	0.527	0.726	54.128	0.242	0.519	51.58	0.318	0.569
cline-cosmic	43.247	0.522	0.723	54.83	0.218	0.515	51.303	0.325	0.575
cline-onehot	43.101	0.526	0.726	55.336	0.209	0.506	51.53	0.319	0.57

### Predicting novel synergistic combinations

In this section, we employed PermuteDDS to predict novel synergistic drug combinations that had not been previously tested. We utilized all measured trios of drug pairs and cell lines to train PermuteDDS and subsequently made predictions for unmeasured trios using the NCI-ALMANAC dataset. We focused on drug combinations with predicted scores close to 1 (see GitHub link^3^.). We further conducted a nonexhaustive literature search, which revealed that six of the predicted drug combinations were consistent with observations from previous studies. For example, Dasatinib and Gefitinib combination presented a cell-specific cytotoxic synergistic effect in human ovarian cell line OVCAR-3 and IGROV-1 [47]. According to the trials of Dolfi et al., combination of fulvestrant and doxorubicin can enhance the sensitivity of breast cancer cell line T47D to these cytotoxic agents [48]. We believe that there are other predicted drug pairs that hold the potential of being promising combinations, which require further validation.

## Discussion

From the results, while PermuteDDS has demonstrated outstanding performance, we noticed that its performance on the leave-cell-out and leave-combination-out tasks is limited. The same situation occurred on other baselines, where $R^{2}$ and PCC scores are consistently below 0.5 and 0.7, respectively. These scores indicate that the predicted results of the model are almost meaningless for these tasks. The reason for this limitation may be attributed to the distribution shift between the training and test sets, caused by the disparity in drug combinations and cell lines between these sets. The problem is expected to be solved by learning the invariance between different drugs and cell lines [49, 50]. Our future work is to explore a more robust model for these leave-out cross validation tasks.

In 3.3, we deduced that the combined use of the three different fingerprints significantly contributed to the predictive performance of PermuteDDS. This observation is likely because these fingerprints provide distinct descriptions of molecules from various perspectives. HashTT fingerprint is a type of path-based fingerprint that incorporates the topological information of molecules [34, 51]. MACCS keys, on the other hand, generate bit strings based on the presence or absence of specific substructures or features, thus enabling the capture of structural information. MAP4 is a novel fingerprint that combines the atom-pair approach with circular substructures, allowing it to encode both molecular shape and chemical information simultaneously [35]. Thus, HashTT provides valuable topological information, while MACCS offers essential structural information. Subsequently, MAP4 supplements the chemical properties from the atomic perspective. The fusion of these distinct pieces of information results in a comprehensive and detailed description of the molecules.

In "Cell line data study" section, we conducted experiments to investigate the significance of cell line descriptors. The results of these experiments suggest that different methods have varying sensitivities to the choice of cell line descriptors, indicating the limitations of one-hot encoding for certain methods. Moreover, the utilization of different cell line descriptors all resulted in poor performance under the leave-cell-out task. Several other related studies have also demonstrated poor generalization performance on this task [52–54]. Nevertheless, we maintain the conviction that continued research in fields such as pharmacology, pharmacokinetics, toxicology, and genetic heterogeneity, alongside the development of novel computational methods, holds the potential to swiftly surmount these challenges.

## Conclusions

In conclusion, we proposed PermuteDDS, a novel model designed to predict potential synergistic drug combinations for cancer treatment. PermuteDDS establishes a unified framework that incorporates diverse types of information, including topological structure and chemical properties of drugs, cellular gene expressions and gene mutations. These different data are effectively fused using the FSN architecture to capture the complex interactions between drug pairs and cell lines. PermuteDDS exhibits robust predictive capabilities on two benchmark datasets through comparison with other competitive methods. However, there remain certain limitations that have been previously discussed. Our future work is to explore a more robust model for leave-out cross validation tasks.

### Supplementary information

**Additional file 1: Table S1. **Performance comparison of one-hot cell line descriptors with different baselines methods on O'Neil dataset.** Table S2.** Performance comparison of one-hot cell line descriptors with different baselines methods on NCI-ALMANAC dataset.

## Data Availability

Lists the following: $\bullet$ Project name: PermuteDDS $\bullet$ Project home page: https://github.com/littlewei-lazy/PermuteDDS $\bullet$ Operating system(s): Linux, Windows $\bullet$ Programming language: Python $\bullet$ Other requirements: Python 3.7 or higher, PyTorch 1.11 or higher

## Abbreviations

HTS: High-throughput screening
SVM: Support vector machine
XGBoost: Extreme gradient boosting
SMILES: Simplified molecular-input line-entry system
CNN: Convolutional neural network
GCN: Graph convolutional network
HashTT: Hashed topological-torsion
MAP4: MinHashed atom-pair fingerprint up to a diameter of four bonds
MACCS: Molecular ACCess System
DDS: Drug-drug synergy
GDSC: Genomics of Drug Sensitivity in Cancer
FSNs: Fingerprint specific networks
HGNN: Hypergraph neural net
PPI: Protein-protein-interaction
Conv1D: One-dimensional convolutional neural network
MSE: Mean square error
RMSE: Root man squared error
$\textrm{R}^2$: Coefficient of determination
PCC: Pearson’s Correlation Coefficient

## Footnotes

1 https://github.com/reymond-group/MAP4/tree/master.

2 https://www.cancerrxgene.org.

3 https://github.com/littlewei-lazy/PermuteDDS/blob/main/Data/novel_synergy_predict.csv
