# Supplementary material for: PermuteDDS: a permutable feature fusion network for drug-drug synergy prediction
Source: J Cheminform. 2024 Apr 15;16:41. doi: 10.1186/s13321-024-00839-8 (PMC11017561; doi:10.1186/s13321-024-00839-8)
Supplement: Supplementary file 1 — Additional file 1: Table S1. Performance comparison of one-hot cell line descriptors with different baselines methods on O'Neil dataset. Table S2. Performance comparison of one-hot cell line descriptors with different baselines methods on NCI-ALMANAC dataset. [file 13321_2024_839_MOESM1_ESM.docx]

**Additional file 1 for PermuteDDS: a permutable feature fusion network for drug-drug synergy prediction**

**1 Performance of one-hot cell line descriptors with different baselines methods**

The results for comparison align with those reported in our manuscript, where the cell line descriptors used are consistent with those reported in the original paper. As depicted in Table S1 and S2, DeepSynergy exhibited a similar phenomenon to PermuteDDS, wherein employing one-hot encoding as a cell line descriptor yielded comparable results to the original results. Especially noteworthy is the performance observed in the leave-cell-out task, where both methods exhibited remarkably poor performance. Even when utilizing fully zero one-hot encoding to represent cell lines, the difference in performance was marginal compared to the original. This observation suggests that it is not only our method that is not sensitive to the selection of cell-line descriptors. However, replacing the cell line descriptor with one-hot encoding leads to a significant decline in the performance of HypergraphSynergy. This may be attributed to the fact that HypergraphSynergy incorporates the reconstruction of cell line similarities as part of its optimization objective. Since the similarity between cell lines encoded using one-hot encoding is uniformly zero, this task becomes meaningless. This observation suggests that not all methods are insensitive to the selection of cell line descriptors, depending on the preprocessing approach applied to the cell lines. Moreover, the experiment results indicate the limitations of one-hot encoding for certain methods.

Table S1 Performance comparison of one-hot cell line descriptors with different baselines methods on O'Neil dataset.

|  | Random split | | |  | Leave cell out | | |  | Leave combination out | | |
| --- | --- | --- | --- | --- | --- | --- | --- | --- | --- | --- | --- |
|  | RMSE | R2 | PCC |  | RMSE | R2 | PCC |  | RMSE | R2 | PCC |
| PermuteDDS | 13.721 | 0.641 | 0.801 |  | 19.668 | 0.243 | 0.522 |  | 16.152 | 0.501 | 0.709 |
| one-hot | 13.663 | 0.644 | 0.803 |  | 20.01 | 0.232 | 0.507 |  | 16.175 | 0.499 | 0.708 |
|  |  |  |  |  |  |  |  |  |  |  |  |
| DeepSynergy | 14.87 | 0.584 | 0.765 |  | 23.89 | 0.195 | 0.426 |  | 17.28 | 0.433 | 0.663 |
| one-hot | 14.624 | 0.592 | 0.77 |  | 21.54 | 0.095 | 0.473 |  | 17.394 | 0.42 | 0.652 |
|  |  |  |  |  |  |  |  |  |  |  |  |
| HyergraphSynergy | 14.727 | 0.586 | 0.775 |  | 19.537 | 0.252 | 0.533 |  | 17.346 | 0.420 | 0.656 |
| one-hot | 19.510 | 0.274 | 0.528 |  | 24.025 | -0.139 | 0.254 |  | 20.793 | 0.169 | 0.420 |

Table S2 Performance comparison of one-hot cell line descriptors with different baselines methods on NCI-ALMANAC dataset.

|  | Random split | | |  | Leave cell out | | |  | Leave combination out | | | |
| --- | --- | --- | --- | --- | --- | --- | --- | --- | --- | --- | --- | --- |
|  | RMSE | R2 | PCC |  | RMSE | R2 | PCC |  | RMSE | R2 | PCC |  |
| PermuteDDS | 43.053 | 0.527 | 0.726 |  | 54.128 | 0.242 | 0.519 |  | 51.58 | 0.318 | 0.569 |  |
| one-hot | 43.101 | 0.526 | 0.726 |  | 55.336 | 0.209 | 0.506 |  | 51.53 | 0.319 | 0.57 |  |
|  |  |  |  |  |  |  |  |  |  |  |  |  |
| DeepSynergy | 44.44 | 0.491 | 0.701 |  | 54.56 | 0.23 | 0.322 |  | 53.5 | 0.262 | 0.526 |  |
| one-hot | 43.013 | 0.528 | 0.727 |  | 55.398 | 0.21 | 0.53 |  | 53.056 | 0.277 | 0.534 |  |
|  |  |  |  |  |  |  |  |  |  |  |  |  |
| HyergraphSynergy | 43.890 | 0.508 | 0.719 |  | 53.398 | 0.273 | 0.538 |  | 52.609 | 0.291 | 0.543 |  |
| one-hot | 52.562 | 0.295 | 0.545 |  | 62.577 | 0.001 | 0.136 |  | 59.046 | 0.107 | 0.333 |  |
